# Color vision and niche partitioning in a diverse neotropical primate community in lowland Amazonian Ecuador

**DOI:** 10.1002/ece3.7479

**Published:** 2021-03-30

**Authors:** Carrie C. Veilleux, Shoji Kawamura, Michael J. Montague, Tomohide Hiwatashi, Yuka Matsushita, Eduardo Fernandez‐Duque, Andres Link, Anthony Di Fiore, Donald Max Snodderly

**Affiliations:** ^1^ Department of Anthropology and Primate Molecular Ecology and Evolution Laboratory University of Texas at Austin Austin TX USA; ^2^ Department of Anatomy Midwestern University Glendale AZ USA; ^3^ Department of Integrated Biosciences University of Tokyo Kashiwa Japan; ^4^ Department of Neuroscience University of Pennsylvania Philadelphia PA USA; ^5^ Department of Anthropology and School of the Environment Yale University New Haven CT USA; ^6^ College of Biological and Environmental Sciences Universidad San Francisco de Quito Cumbayá Ecuador; ^7^ Department of Biological Sciences Universidad de Los Andes Bogota Colombia; ^8^ Department of Neuroscience University of Texas at Austin Austin TX USA

**Keywords:** color vision, community ecology, intraspecific variation, niche partitioning, opsin gene, primate

## Abstract

A recent focus in community ecology has been on how within‐species variability shapes interspecific niche partitioning. Primate color vision offers a rich system in which to explore this issue. Most neotropical primates exhibit intraspecific variation in color vision due to allelic variation at the middle‐to‐long‐wavelength opsin gene on the X chromosome. Studies of opsin polymorphisms have typically sampled primates from different sites, limiting the ability to relate this genetic diversity to niche partitioning. We surveyed genetic variation in color vision of five primate species, belonging to all three families of the primate infraorder Platyrrhini, found in the Yasuní Biosphere Reserve in Ecuador. The frugivorous spider monkeys and woolly monkeys (*Ateles belzebuth* and *Lagothrix lagotricha poeppigii*, family Atelidae) each had two opsin alleles, and more than 75% of individuals carried the longest‐wavelength (553–556 nm) allele. Among the other species, *Saimiri sciureus macrodon* (family Cebidae) and *Pithecia aequatorialis* (family Pitheciidae) had three alleles, while *Plecturocebus discolor* (family Pitheciidae) had four alleles—the largest number yet identified in a wild population of titi monkeys. For all three non‐atelid species, the middle‐wavelength (545 nm) allele was the most common. Overall, we identified genetic evidence of fourteen different visual phenotypes—seven types of dichromats and seven trichromats—among the five sympatric taxa. The differences we found suggest that interspecific competition among primates may influence intraspecific frequencies of opsin alleles. The diversity we describe invites detailed study of foraging behavior of different vision phenotypes to learn how they may contribute to niche partitioning.

## Graphical abstract

Neotropical primates exhibit substantial variation within and between species in color vision abilities; however, little work has explored interspecific variation in a single primate community. In this study, we genotyped functional variation in the middle‐to‐long‐wavelength opsin gene in populations of five sympatric primate species at the Yasuní Biosphere Reserve in Ecuador. We found substantial interspecific and intraspecific variation in color vision across the five species and discuss our findings in the context of dietary specialization and niche partitioning.

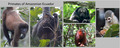

## 1 INTRODUCTION

Ecologists have long been interested in how sympatric species coexist and partition ecological niche space (Gause, 1934; Hutchinson, 1957; Macarthur & Levins, 1967). Sympatric primates, for example, may partition niche space through differences in diet, microhabitat utilization, and/or timing of activity (Ganzhorn, 1989a, 1989b; Schoener, 1974; Snodderly et al., 2019; Terborgh, 1984; Youlatos, 1999). Primatologists have sought to identify morphological and behavioral adaptations in sympatric species that might be associated with resource partitioning (Norconk et al., 2009; Rosenberger, 1992; Youlatos & Meldrum, 2011), including adaptations for exploiting fallback foods in times of scarcity (Hemingway & Bynum, 2005; Lambert et al., 2004; Terborgh, 1984), and locomotor adaptations associated with microhabitat usage (Rodman, 1979). As part of this effort, sensory systems can be particularly important, as they represent some of the primary ways animals interact with their environments. Interspecific differences in sensory function can allow sympatric species to coexist by exploiting different foods, microhabitats, or other aspects of their environments (Leal & Fleishman, 2002; Siemers & Swift, 2006; Smith, 2000). In addition, intraspecific variation in sensory function can expand the range of resources that a given species can efficiently utilize (Melin et al., 2007; Smith et al., 2012), broadening its niche, and potentially improving its competitiveness.

In relation to niche partitioning, color vision has been one of the best‐studied senses due to the ecological importance of vision and the clear genotype–phenotype relationship between vision genes and spectral sensitivity of retinal photoreceptors (Hauser & Chang, 2017; Saito et al., 2005). The role of color vision variation in niche partitioning is particularly apparent in sympatric fish species (Hofmann et al., 2009; Nandamuri et al., 2017; Stieb et al., 2017), and interspecific differences in cone spectral sensitivity have also been linked to ecological niche characteristics in some terrestrial taxa. For example, variation in cone spectral sensitivities among sympatric *Anolis* lizards is associated with the ambient light environments of their microhabitats (Leal & Fleishman, 2002), while spectral tuning of short‐wavelength‐sensitive cones has been linked to fruit and flower consumption in nocturnal mammals (Veilleux & Cummings, 2012).

Neotropical primates represent a good group in which to relate color vision variation to ecological diversity and niche partitioning. Following their arrival in Central and South America in the mid‐ to late Eocene, primates experienced an adaptive radiation into open ecological niche space (Aristide, Rosenberger, Tejedor, & Perez, 2015; Kiesling, Yi, Xu, Gianluca Sperone, & Wildman, 2015; Seiffert et al., 2020; Silvestro et al., 2019). Modern neotropical community assemblages—particularly those at relatively undisturbed sites in the Amazon and Orinoco basins—typically include multiple sympatric species that vary in their use of food resources (Dew, 2005; Rosenberger, 1992; Stevenson et al., 2000; Terborgh, 1984), locomotor strategies (Youlatos & Meldrum, 2011), and use of vertical strata within the forest (Fleagle & Mittermeier, 1980; Sheth et al., 2009; Youlatos, 1999).

The broad ecological distribution of neotropical primates is accompanied by an array of highly variable color vision systems. Most taxa have a single middle‐to‐long‐wavelength (M/L) opsin gene locus on the X chromosome and a short‐wavelength (S) autosomal locus. Single nucleotide polymorphisms (SNPs) at critical sites in the M/L opsin gene can result in M/L opsin proteins with differing spectral sensitivities (Hiramatsu et al., 2004; Kawamura, 2018; Matsumoto et al., 2014; Yokoyama et al., 2008). Females homozygous at the X‐linked M/L locus and all males express only one type of M/L opsin protein in their retinal cones and thus exhibit dichromatic color vision. Females that are heterozygous at the M/L locus express two different M/L opsin proteins in their cones, which, in conjunction with the autosomal S cone, confer “polymorphic trichromacy” (Jacobs, 2008; Veilleux, 2017). Consequently, most neotropical primate taxa exhibit a diversity of color vision phenotypes. Two genera deviate from this pattern: *Alouatta* (family Atelidae) and *Aotus* (family Cebidae). *Alouatta* has evolved “routine” trichromatic color vision due to juxtaposition of two alleles of the ancestral M/L opsin gene (Jacobs et al., 1996; Matsumoto et al., 2014), such that all individuals are trichromats. By contrast, the nocturnal/cathemeral genus *Aotus* has one M/L opsin allele and has also lost function in the S opsin gene, resulting in monochromatic color vision (Jacobs et al., 1993; Mundy et al., 2016).

A recent focus in community ecology has been examining the importance of intraspecific variability for understanding niche overlap and species coexistence (Bolnick et al., 2011; Violle et al., 2012). While most neotropical primates exhibit polymorphic trichromacy, prior research suggests that species can differ dramatically in the number of spectrally distinct M/L opsin alleles they possess. For example, non‐*Alouatta* atelids (e.g., *Ateles*, *Lagothrix*) typically exhibit only two alleles (Hiramatsu et al., 2005; Matsumoto et al., 2014), most cebids (e.g., *Saguinus*, *Saimiri*, *Cebus*, except *Aotus*) have three alleles (Hiramatsu et al., 2005; Jacobs et al., 1993; Kawamura & Melin, 2017; Surridge et al., 2005), and pitheciids (e.g., *Plecturocebus* [formerly *Callicebus*], *Pithecia*, *Cacajao*, *Chiropotes*) can have between three and six alleles (Bunce et al., 2011; Corso et al., 2016; Goulart et al., 2017; Jacobs & Deegan, 2005). This variation in the number of spectrally different M/L alleles establishes a great diversity of color vision phenotypes, which potentially facilitate the exploitation of a diversity of resources (Melin et al., 2014).

Using a coalescence simulation study, Hiwatashi et al. (2010) compared the nucleotide diversity and the nucleotide configuration spectrum of the M/L opsin gene to those of neutral genome regions sampled from the same populations of *Ateles geoffroyi* and *Cebus imitator* in Costa Rica. Based on their simulations, they rejected a neutral, drift‐based null model for the maintenance of polymorphism at the M/L opsin locus and instead found support for a balancing selection model. Given this study and the long evolutionary history and more or less finite population sizes of platyrrhines over that time, the existence of high allelic polymorphism at the M/L opsin locus in almost every study population of wild platyrrhines is reasonably regarded as empirical evidence for balancing selection surpassing the effect of random genetic drift in explaining contemporary patterns of M/L opsin variation. Thus, we are in a stage where it is justified to consider possible explanations based on balancing selection.

**FIGURE 1:**
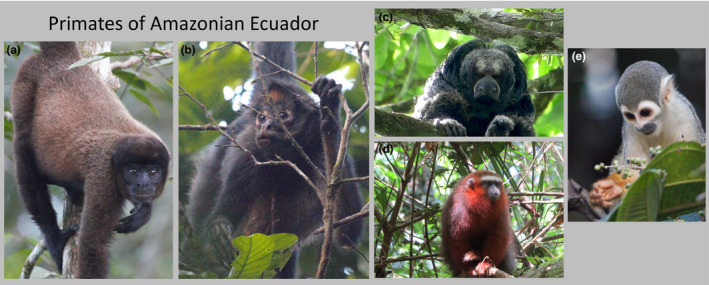
Species included in this study. (a) *Lagothrix lagotricha poeppigii*, (b) *Ateles belzebuth*, (c) *Pithecia aequatorialis*, (d) *Plecturocebus discolor*, and (E) *Saimiri sciureus macrodon*. Photo credits for (a), (b), and (e): Sam Hibdige. Photo credits for (c) and (d): Anthony Di Fiore

Although variation in color vision among neotropical primates has been extensively documented, with rare exceptions—for example, *A. geoffroyi* and *C. imitator* in Costa Rica (Hiramatsu et al., 2005; Hiwatashi et al., 2010) and *Saguinus fuscicollis* and *S. mystax* in Peru (Surridge et al., 2005)—studies have typically examined opsin polymorphism in only one species at a given field site. We examined intraspecific and interspecific variation in color vision of sympatric primate species at the Tiputini Biodiversity Station and the nearby Proyecto Primates Research Area (Di Fiore et al., 2009) in Amazonian Ecuador. The primate community in this region includes 10 species from 10 different genera, nine of which are diurnal (Marsh, 2004). We collected data for five of the diurnal species (Table 1; Figure 1). These taxa include representatives of all three major evolutionary lineages of neotropical primates.

**TABLE 1:** Ecological characteristics and numbers of study subjects for the five sympatric primate taxa in this study

Species	Common name	Body mass (kg)^a^	Dietary preferences^b^	Mean forest height (m)^c^	Samples in this study		
					Males	Females	Social groups
*Ateles belzebuth*	White‐bellied spider monkeys	9.0–9.3	Fruit, leaves^1^, ^2^	22.5	3	5	2
*Lagothrix lagotricha poeppigii*	Lowland woolly monkeys	5.5–7.5	Fruit, leaves, prey^1^, ^3^	21.9	3	6	3
*Plecturocebus (Callicebus) discolor*	Red titi monkeys	0.8–0.9	Fruit, leaves, prey^4^	10.6	8	8	6
*Pithecia aequatorialis*	Equatorial saki monkeys	2.0–2.6	Fruit and seeds^5^	19.1	5	4	4
*Saimiri sciureus macrodon*	Squirrel monkeys	0.7–0.8	Prey and fruit^6^	12.4	28	34	4

^a^ Body mass data (female–male) for *Ateles*, *Lagothrix*, *Plecturocebus*, and *Pithecia* at Tiputini (Snodderly et al., 2019). *Saimiri* data from Smith and Jungers (1997).

^b^ Based on items comprising more than 4% of the diet, in decreasing order of feeding or foraging time.

^c^ Mean observed height in the forest for our study taxa at Tiputini (Sheth et al., 2009).

^1^ Data from Yasuní National Park, Ecuador, ~35 km from Tiputini.

^2^ Dew (2005), Di Fiore et al. (2008), Link and Di Fiore (2006).

^3^ Dew (2005), Di Fiore (2004).

^4^ Detailed dietary data for *P*. *discolor* are not currently available; these preferences are based on closely related *Plecturocebus cupreus* *ornatus* in Macarena, Colombia (Bicca‐Marques & Heymann, 2013).

^5^ Data for *P. aequatorialis* from A. Di Fiore et al. (unpublished data, 2015‐2019).

^6^ Data from Tiputini (Montague, 2011).

Typical of primates, our study taxa utilize their environment flexibly, and they occupy broad, overlapping ecological niches (Table 1). Home ranges of the different species are spatially superimposed, and all use upland forests extensively, though there may be differential, less‐extensive use of swampy and periodically water‐logged areas with high densities of certain palms (Sheth et al., 2009). Within the forest, larger‐bodied species tend to occupy higher strata than smaller‐bodied ones (Sheth et al., 2009). While our study species represent a range of diverse diets and lifestyles, they overlap in their consumption of ripe fruit, a key dietary resource. For example, the eight plant genera that comprise 72% of the fruit feeding trees used by *Saimiri sciureus macrodon* also comprise 15% and 26% of the fruit feeding observations for *Ateles belzebuth* and *Lagothrix lagotricha poeppigii*, respectively (Dew, 2005; Montague, 2011). The spatial and dietary overlap among primate taxa can result in agonistic interactions at fruiting trees, in which larger‐bodied species displace smaller‐bodied species, and large social groups displace small social groups (Bicca‐Marques & Heymann, 2013; D.M. Snodderly & A. Di Fiore, personal observations 2015‐2019). In such cases, the smaller monkeys may benefit from niche partitioning that includes the utilization of other food sources that have different visual properties. Here, we document the diversity in color vision alleles and phenotypes in these sympatric primates that may contribute to niche partitioning.

## 2 MATERIALS AND METHODS

### 2.1 Study sites

The Tiputini Biodiversity Station is located in primary lowland rainforest along the left bank of the Río Tiputini—a major tributary of the Río Napo that feeds into the Amazon—and adjacent to the 980,000 ha Yasuní National Park in eastern Ecuador (76°08′W, 0°38′S; Bass et al., 2010). The research station occupies ~744 ha of protected land, 90% of which is unflooded *terra firme* forest. The forest is evergreen and there is no pronounced dry season, but there is about a twofold seasonal variation in rainfall and in the availability of ripe, fleshy fruit (Snodderly et al., 2019). The Proyecto Primates Research Area (Di Fiore et al., 2009) is a similarly forested habitat located within the Yasuní National Park, ~35 km to the west of Tiputini and well inland from any large rivers. Samples of all individuals of the genera *Lagothrix*, *Plecturocebus*, *Pithecia*, and *Saimiri* were collected at Tiputini, as were four samples from *Ateles*. Four additional *Ateles* samples were collected at the Proyecto Primates Research Area.

### 2.2 Biological samples

Fecal samples were collected opportunistically from identified *Ateles* and unidentified *Lagothrix* individuals between 2003 and 2006, and from both identified and unidentified individuals of *Saimiri* between 2006 and 2008. Samples were preserved at room temperature in RNAlater (Invitrogen) nucleic acid preservation buffer. Tissue samples were collected between 2003 and 2016 from known individuals of *Pithecia* and *Plecturocebus* who had been captured to affix radio collars to them and to collect biometric data. For all species, samples were confirmed to come from different individuals based on their unique SSR genotypes across a panel of hypervariable markers (seven to 12 loci, depending on the species; Montague et al., 2014). For all species, samples were collected from more than one social group. All animal capture and sample collection protocols followed guidelines for the International Primatological Society's Code of Best Practices for Field Primatology (2014) and were approved by the University Animal Welfare Committee at New York University (protocol numbers: UAWC #01‐1103, #04‐1217, #04‐1218, #05‐1250, #05‐1252, and #06‐1266) and the Institutional Animal Care and Use Committee at The University of Texas at Austin (protocol numbers: AUP 2011‐0146 and AUP‐2014‐00411). Sampling and fieldwork were authorized by the Ecuadorian Ministry of the Environment.

### 2.3 Genotyping analyses

#### 2.3.1 DNA extraction and sequencing

We extracted genomic DNA from tissue and fecal samples of all species using Qiagen DNeasy Blood and Tissue Kits and QIAmp Stool Mini Kits, respectively. In primates, variation in M/L opsin tuning is primarily determined by amino acid sites in exons 3 and 5 of the M/L opsin gene (Hiramatsu et al., 2005; Jacobs et al., 2017; Yokoyama et al., 2008). Consequently, we focused attention on exons 3 and 5 in this study. For *Ateles*, *Lagothrix*, *Plecturocebus*, and *Pithecia*, we determined opsin genotypes for individual samples by sequencing exons 3 and 5. We thus used the polymerase chain reactions (PCRs) to amplify exons 3 and 5 in each individual (PCR conditions and primer sequences are provided in Supporting Information). For *Plecturocebus* and *Pithecia* samples, PCR products were separated and sequenced at The University of Texas at Austin on an Applied Biosystems 3730/3730XL DNA Analyzer, and the chromatograms were analyzed using the software Geneious v 9.0.5 (https://www.geneious.com). Similarly, PCR products for *Ateles* and *Lagothrix* samples were separated and sequenced on an Applied Biosystems 3130 DNA Analyzer at the University of Tokyo, and the chromatograms were analyzed in Applied Biosystems “Sequencing Analysis 5.2” or Geneious version 9.0.5 (Kearse et al., 2012).

#### 2.3.2 M/L opsin genotyping

**FIGURE 2:**
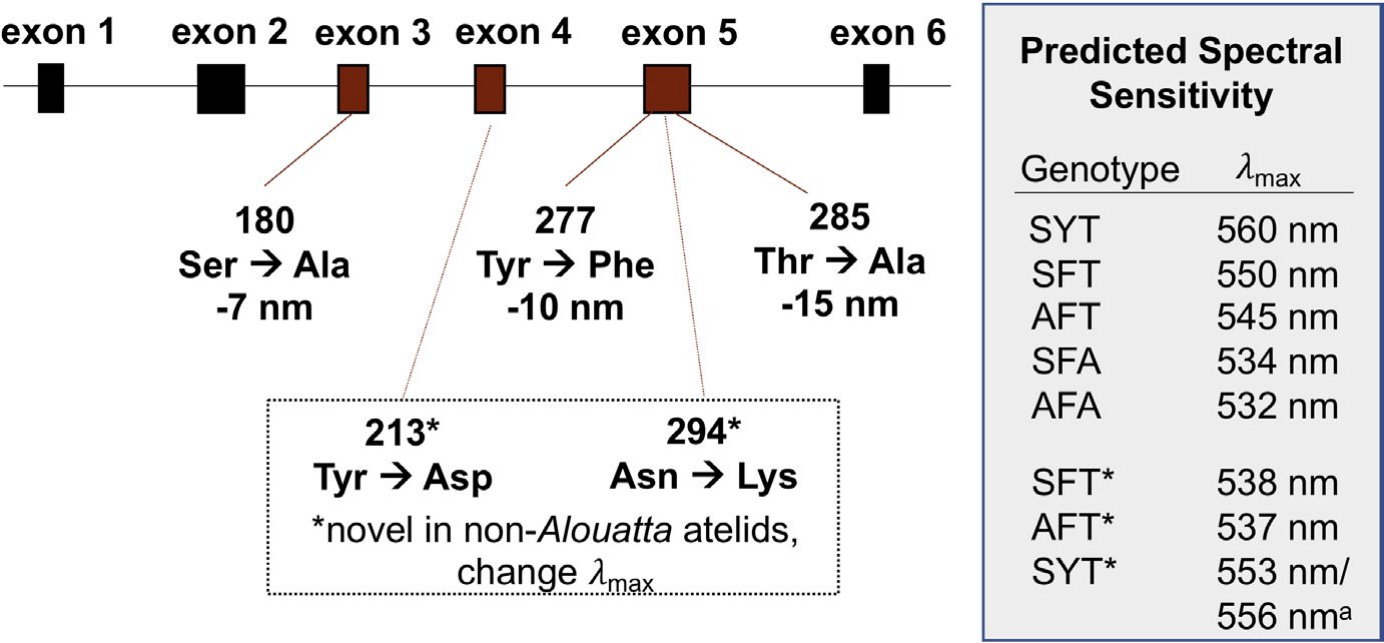
Middle‐to‐long‐wavelength (M/L) opsin gene spectral tuning. The “three‐sites rule” predicts the spectral tuning of primate M/L opsins based on known effects of amino acid substitutions at sites 180, 277, and 285 on opsin pigment *λ*_max_. Recent work (Matsumoto et al., 2014) identified novel substitutions in non‐*Alouatta* atelids (*Ateles*, *Lagothrix*) that result in opsin pigment *λ*_max_ that deviates from predictions of the three‐sites rule (*). ^a^Pigment reconstitution experiments further demonstrate that *λ*_max_ of the SYT allele differs between *Ateles* and *Lagothrix* (Matsumoto et al., 2014). Figure adapted and modified from Hiramatsu et al. (2005)

The spectral sensitivity of primate M/L opsins can be predicted primarily using the residues at three M/L opsin amino acid sites (Figure 2): position 180 (located in exon 3) and positions 277 and 285 (both located in exon 5). Consequently, most studies of platyrrhine color vision infer the tuning of M/L opsin alleles based on the so‐called “three‐sites rule” (Bunce et al., 2011; de Lima et al., 2015; Goulart et al., 2017; Hiramatsu et al., 2005). However, Matsumoto et al. (2014) recently identified two novel nonsynonymous mutations (in amino acids 213 and 294) that shift the spectral sensitivities of M/L opsins more than predicted by the three‐sites rule (Figure 2). These two mutations evolved in the last common ancestor of non‐*Alouatta* atelids, and they are found in both *Ateles* and *Lagothrix* in Ecuador. The two M/L opsin alleles of non‐*Alouatta* atelids share aspartic acid at site 213, while the two alleles are segregated into asparagine and lysine at site 294 (Matsumoto et al., 2014). We used the “three‐sites rule” and residue 294 to distinguish alleles of *Ateles*, *Lagothrix*, *Plecturocebus*, and *Pithecia* individuals. In addition, we sequenced exon 4 in one of the female *Ateles* and confirmed the presence of aspartic acid at site 213. Three females (two *Pithecia* one *Plecturocebus*) were heterozygous at more than one of the three spectral tuning sites. To estimate the separate alleles for the heterozygous females, we used SeqPhase (Flot, 2010) to transform *fasta* alignments into input files for the statistical haplotyping program PHASE v.2.1.1 by which all the mathematically possible haplotypes are tested whether they meet the Hardy–Weinberg equilibrium to estimate the true haplotypes (Stephens & Donnelly, 2003; Stephens et al., 2001).

We employed an alternate genotyping method for *Saimiri* individuals. In *Saimiri*, each of the alternative amino acids present at position 180 (serine or alanine) is consistently associated with a particular amino acid at position 277 (tyrosine or phenylalanine, respectively; Cropp et al., 2002; Rowe & Jacobs, 2004). This pattern makes position 277 redundant with position 180, allowing genotypes (and the corresponding visual system phenotype) at the “three‐sites” to be determined by resolving only the identity of the amino acids at positions 180 and 285. Therefore, we genotyped the *Saimiri* samples by interrogating just two of the three spectral tuning sites—positions 180 and 285—using a custom‐designed TaqMan^®^ (Applied Biosystems) assay for each of these sites. The procedure allows fluorescent oligonucleotide probes to complement and bind to either one or both of the potential SNP sites at positions 180 and 285 in each sample of DNA extracted from feces. In samples of males and homozygous females, only one of the two probes binds to the SNP site at each of these positions. Detailed description of the genotyping PCR conditions is provided in Supporting Information (Materials and Methods S2).

### 2.4 Statistical testing and limitations

We evaluated the degree of uniformity of allele distributions and of differences between allele distributions with the chi‐squared tests in R, version 3.5.2 (R Core Team, 2020). As in most observational studies, the samples we have for analysis do not represent a random sample from the population. Consequently, the assumptions of the chi‐square test cannot be fully satisfied. We also know that some individuals sampled are related to one another and therefore their samples are not fully independent. However, they are part of the local ecology and participate in competitive interactions with one another and with individuals of other species; thus, it is important to include them. In every case where we report the results of an inferential test, we also present the appropriate data so that the magnitude of the differences can be considered; the chi‐square test provides an additional commonly used measure for interpreting the probability of having obtained the reported differences.

## 3 RESULTS

We analyzed the M/L opsin gene on 161 X chromosomes of 104 individuals (47 males and 57 females: Table 1), using either the Sanger sequencing or SNP genotyping assays as described above. Exons 3 and 5 were examined for all individuals; exon 4 was also sequenced for one of the female spider monkeys as reported in a prior publication (Matsumoto et al., 2014). For *Plecturocebus*, *Pithecia*, and *Saimiri*, we predicted peak spectral sensitivity (*λ*_max_) of the M/L alleles(s) of each individual based on the amino acids at residues 180, 277, and 285 following the “three‐sites rule” and results of previously published reconstitution experiments (Table 2). For the atelids (*Ateles* and *Lagothrix*), our predictions of peak spectral sensitivity included the effect of the amino acid at site 294; for these predictions, we assumed that all non‐*Alouatta* atelids had aspartic acid at site 213 in exon 4, as the Y213D mutation in the common ancestor of the atelids predates the appearance of the two current opsin alleles (Matsumoto et al., 2014).

**TABLE 2:** M/L opsin alleles and peak wavelength (*λ*_max_) of the corresponding opsin grouped by genotype and sex

Taxon	Alleles^a^	Opsin *λ*_max_ (nm)^b^	No. of male dichromats	No. of female dichromats	Female trichromats		Total no. of individual sampled
					Alleles (*λ*_max_ ^c^)	No.	
*Ateles*	SFT	538	2	—	538 + 553 (15)	3	8
	**SYT**	553	1	2			
*Lagothrix*	AFT	537	—	1	537 + 556 (19)	2	9
	**SYT**	556	3	3			
*Plecturocebus*	AFA	532	2	—	545 + 550 (5)	1	
	**AFT**	545	3	3	550 + 560 (10)	2	16
	SFT	550	2	—		1	
	SYT	560	1	1	545 + 560 (15)		
*Pithecia*	AFA	532	1	—	532 + 545 (13)	2	
	**AFT**	545	3	—	545 + 560 (15)	1	9
	SYT	560	1	—	532 + 560 (28)	1	
*Saimiri*	AFA	532	2	1	532 + 545 (13)	4	
	**AFT**	545	16	10	545 + 558 (13)	11	62
	SYT	558	10	5	532 + 558 (26)	3	

^a^ Letters correspond to amino acids at sites 180, 277, and 285. The most common allele for each taxon is bolded. Note: For *Ateles/Lagothrix*, we assumed site 213 was aspartic acid for all individuals; at site 294, SYT allele had asparagine (N), while SFT and AFT alleles had lysine (K).

^b^ For *Ateles*, *Lagothrix*, and *Saimiri*, *λ*_max_ was determined by reconstitution of the pigments of representative individuals with these genotypes (Hiramatsu et al., 2004; 2008; Matsumoto et al., 2014).

^c^ Value in parentheses represents the estimated difference in nanometers between the two opsin pigments.

### 3.1 Numbers of alleles and variations in allele frequencies

**FIGURE 3:**
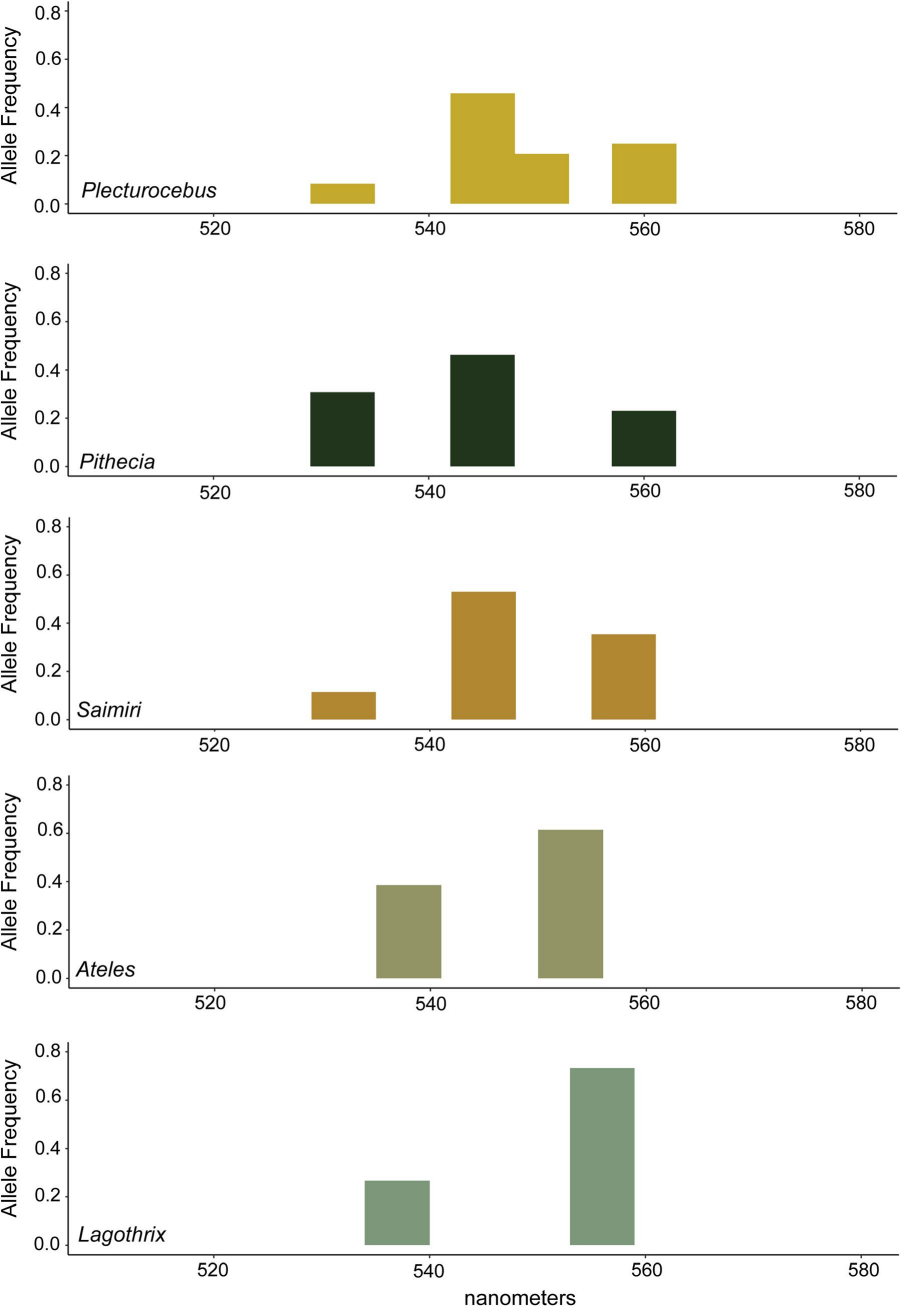
Allele frequencies for the middle‐to‐long‐wavelength opsin gene for each species sampled. Wavelength of alleles (in nanometers) on the *x*‐axis. Allele wavelengths following Figure 1: AFA (532 nm), AFT* (*Ateles*: 537 nm), SFT* (*Lagothrix*: 538 nm), AFT (545 nm), SFT (550 nm), SYT* (*Ateles*: 553 nm; *Lagothrix*: 556 nm), and SYT (560 nm)

We found substantial variation in numbers and relative frequencies of M/L opsin alleles across taxa (Figure 3; Table 2). The non‐*Alouatta* atelids each had only two alleles (*Ateles*: SFT and SYT; *Lagothrix*: AFT and SYT). By contrast, we detected three alleles in *Saimiri* and *Pithecia* (AFA, AFT, and SYT) and four alleles in *Plecturocebus* (AFA, AFT, SFT, and SYT). These allele types (i.e., three amino acid haplotypes) were directly determined from male or homozygous female samples. The presence of recombinant alleles between them in heterozygous females was not supported by our PHASE analysis. The most common allele also differed across taxa. The SYT allele, for example, encodes the opsin with the most red‐shifted *λ*_max_ in both the non‐atelids (*λ*_max_ 558–560 nm) and the atelids (*λ*_max_ 553–556 nm). While the SYT allele was present in all species, the relative frequency was highly variable (Figure 3). In the atelids, the frequency of the SYT allele was 62% in *Ateles* and 73% in *Lagothrix* (Figure 3). Considering *Ateles* and *Lagothrix* together, the SYT allele occurred in 82% of atelid individuals. In the non‐atelids, however, the SYT allele occurred in less than half of individuals, and the relative frequency of the allele was substantially lower: 25% in *Plecturocebus*, 23% in *Pithecia*, and 35% in *Saimiri*. Instead, the most common allele in the three non‐atelid species (Figure 3) was the mid‐wavelength AFT allele (*λ*_max_ of 545 nm) at 46% (*Plecturocebus*), 46% (*Pithecia*), and 53% (*Saimiri*). The relatively small sample for *Pithecia* does not warrant an inferential statistical test, but we found that distributions of opsin alleles differed from a uniform distribution for both *Plecturocebus* ($\chi_{2}^{2}$ = 7.01, *p* =.030) and *Saimiri* ($\chi_{2}^{2}$ = 25.2, *p* <.001).

### 3.2 Genotype frequencies and numbers of dichromats and trichromats

**FIGURE 4:**
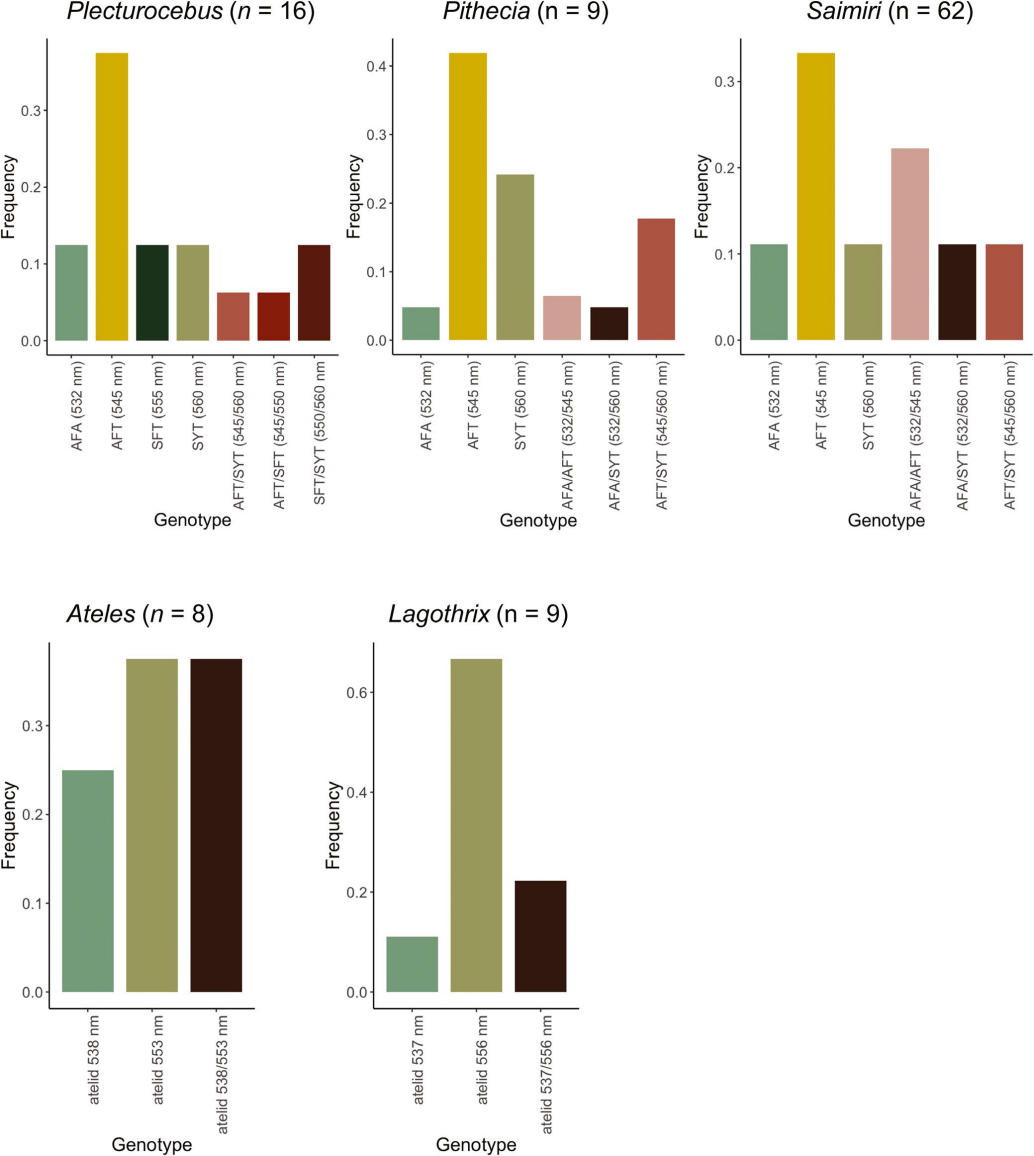
Frequencies of middle‐to‐long‐wavelength (M/L) opsin genotypes per species. Dichromats have only one M/L opsin along with an S opsin (not indicated). Trichromats have two M/L opsins along with an S opsin. Numerical details are in Table 2

We identified both trichromatic and dichromatic genotypes in all five taxa (Figure 4). Each taxon had at least one dichromat with each of the detected alleles, contributing to intraspecific diversity of genotypes. Consistent with the allele frequency results, the most common dichromatic genotype among both non‐*Alouatta* atelids was the long‐wavelength SYT allele (Figure 4; Table 2). In contrast, the most common dichromatic genotype among all three non‐atelids had the mid‐wavelength AFT allele, again consistent with allele frequencies.

The proportion of females that were trichromats varied across taxa (Table 2), ranging between 22% (*Lagothrix*) and 37% in *Ateles* to 100% (*Pithecia*); the other two species ranged around 50% (*Plecturocebus*—50% and *Saimiri*—53%). However, sample sizes were limited, and for *Pithecia*, three of the sampled females were related. Thus, any interspecific differences must be interpreted with caution. Only one trichromatic genotype was possible in the two atelid taxa because their gene pools only included two M/L opsin alleles. However, *Saimiri* and *Pithecia* each had three segregating alleles, allowing for three possible trichromatic genotypes, and we detected individuals of all three types for both species. The distribution of trichromatic genotypes in *Saimiri* was nonuniform ($\chi_{2}^{2}$ = 6.35, *p* =.042), and the most common genotype was AFT/SYT (545/558, 13 nm difference), found in 11 out of 18 trichromat females. With four alleles, *Plecturocebus* could theoretically exhibit as many as six different trichromatic genotypes; we identified individuals of three of these types in the Tiputini sample of 8 female individuals. Although the most common dichromatic genotype (AFT allele, 545 nm) was shared by all non‐atelid species, they appear to differ in the most common trichromatic genotype (Figure 4). However, additional data will be needed to adequately characterize the distributions of trichromats.

## 4 DISCUSSION

The sympatric primates at Tiputini occupy broad, overlapping, multidimensional ecological niches (Table 1). On the basis of our results, we suggest that variation in color vision should be included as one of the traits that may contribute to niche partitioning. We focus here on possible relationships between variation in color vision and differences in food choice and foraging behavior within and across taxa. Particular color vision genotypes should yield advantages in detecting and utilizing particular food sources, which could contribute to establishing niches biased toward utilization of those resources. At the same time, other factors, such as interspecific competition, may limit access to preferred resources, thereby favoring different vision genotypes better suited to utilizing alternative resources. Thus, variation in color vision within and across species may dynamically contribute to niche partitioning and might do so in a manner that varies with geographic location and with the composition of the local ecological community. Here, we describe the color vision genetics of our study sample and compare our results to data from other sites with different primate community compositions and habitat types.

Our study is unique in characterizing the diversity of color vision in five genera of sympatric primates living in a hyperdiverse primary lowland rain forest (Bass et al., 2010). These data represent the first genetic determinations of opsin alleles in wild *Pithecia aequatorialis* and *Plecturocebus discolor*. They are also the first population data for Ecuadorian *A. belzebuth*, *L. lagotricha poeppigii*, and *S. sciureus macrodon*. All the individual alleles that we detected had been reported previously for the genera we have studied: *Ateles* and *Lagothrix* (Hiramatsu et al., 2005; Matsumoto et al., 2014), *Pithecia* (Boissinot et al., 1998), *Plecturocebus*/*Callicebus* (Bunce et al., 2011; Goulart et al., 2017), and *Saimiri* (Cropp et al., 2002; Hiramatsu et al., 2004; Neitz et al., 1991; Rowe & Jacobs, 2004).

### 4.1 Number and relative frequency of opsin alleles

#### 4.1.1 Atelids

Our sample of 17 Ecuadorian non‐*Alouatta* atelids yielded only two M/L opsin alleles for each species: SFT and SYT for *Ateles* and AFT and SYT for *Lagothrix*. In each species, the longer‐wavelength SYT allele was more frequent. The pattern of two alleles per species, with a bias toward the SYT allele, is consistent with genetic data from 32 *A. geoffroyi* from Costa Rica (Hiramatsu et al., 2005; Hiwatashi et al., 2010) and from 18 muriquis (13 *Brachyteles arachnoides* with SFT and SYT and five *Brachyteles hypoxanthus* with AFA and SYT) from Brazil (Talebi et al., 2006). The limitation to two alleles is also consistent with electroretinographic (ERG) results from 56 non‐*Alouatta* atelids housed in captive colonies (18 *A. geoffroyi*, 12 *Ateles fusciceps robustus*, 17 potential *Ateles* hybrids, and 9 *L. lagotricha poeppigii,* which likely included animals from Ecuador and Colombia; Jacobs & Deegan, 2005). In total, accumulated data from 123 individuals from the three non‐*Alouatta* atelid genera exhibit a clear pattern for this clade: For each species, there is a single X‐linked gene with only two opsin alleles. Where genetic data are available, all species are found to have the long‐wavelength SYT allele, which is present at a higher frequency than the alternate, middle‐wavelength allele, which differs from species to species.

#### 4.1.2 Non‐atelids: *Plecturocebus*, *Pithecia*, and *Saimiri*

We observed a very different pattern in the three non‐atelid genera. All three species were polymorphic, with either three (*Pithecia*, *Saimiri*) or four (*Plecturocebus*) opsin alleles. Each of the three species also exhibited a bias toward the middle‐wavelength AFT allele instead of the long‐wavelength SYT allele.

##### Plecturocebus

We detected the largest number of opsin alleles (AFA, AFT, SFT, SYT) in titi monkeys, *P. discolor* (family Pitheciidae). An additional allele, for a total of five, has previously been identified by ERG measurements in a large captive population (*n* = 82) that was initially described as *Callicebus moloch* (Jacobs & Deegan, 2005). The species composition of this captive colony was later clarified to consist of representatives of *Plecturocebus cupreus cupreus*, *P*. *cupreus ornatus*, and possible hybrids (Bunce, 2009; Bunce et al., 2011). Consequently, it is an open question whether the allele count for the captive population (five alleles) represents the true opsin diversity expected for wild *Plecturocebus* populations, particularly if there is any interspecific variation in the number of alleles. The only previous study of a wild population of *Plecturocebus* (*P. toppini*, in Peru, previously called *Callicebus brunneus*) detected only three alleles, with the most common being AFT (Bunce et al., 2011), similar to our results for *P. discolor*. Thus, our results are the first unequivocal evidence for the presence of more than three opsin alleles in any wild population of *Plecturocebus*. Notably, our sample size is still relatively small (*n* = 16), and studies of wild populations should be expanded to determine the full opsin gene diversity in *Plecturocebus* in natural breeding populations.

##### Pithecia

We identified three opsin alleles in *P. aequatorialis* (AFA, AFT, and SYT). These same alleles were also found previously in 16 male captive *P. irrorata* in Brazil (Boissinot et al., 1998). A more recent study (Goulart et al., 2017) that included museum specimens of two female *P*. *irrorata* identified a single novel allele (AYT), indicating that *P. irrorata* has four opsin alleles. This AYT allele has not yet been detected in the Tiputini primates. Similar to *Plecturocebus*, AFT was the most common allele among Tiputini *P. aequatorialis*. Our study is the first to report the M/L opsin gene frequencies for a wild population of the genus *Pithecia*.

##### Saimiri

For *Saimiri*, we employed a SNP genotyping approach to explore allelic variation in the Tiputini population. A large survey of genetic data identified only three alleles (AFA, AFT, and SYT) in a sample of 362 X chromosomes from multiple *Saimiri* species and locales (Rowe & Jacobs, 2004; Table 3). Additionally, only two exceptional cases of recombinant alleles have been found in *Saimiri*: one in *S. boliviensis*, with an intermediate predicted *λ*_max_ of 558 nm, and another one in *S. sciureus*, with a predicted *λ*_max_ of 534 nm (Cropp et al., 2002). These results suggest that alleles other than the major three are exceptionally rare in *Saimiri*, supporting our use of the SNP genotyping approach in lieu of complete sequencing of multiple opsin exons.

**TABLE 3:** Middle‐to‐long‐wavelength opsin allele frequencies in *Saimiri* populations

Species	AFA	AFT	SYT	Data
*S. boliviensis*	24 (26.1)	41 (44.6)	27 (29.3)	Cropp et al. (2002)
*S. oerstedii*	14 (20.6)	25 (36.8)	29 (42.6)	Cropp et al. (2002)
*S. sciureus*	29 (29.9)	36 (37.1)	32 (33.0)	Cropp et al. (2002)
*S. s. macrodon*	11 (11.5)	51 (53.1)	34 (35.4)	This study
Pooled data	106 (29.3)	124 (34.3)	132 (36.5)	Rowe and Jacobs (2004)

Note

Pooled data include data from Cropp et al. (2002), as well as all other sources compiled by Rowe and Jacobs (2004). They do not include data from this study. Allele counts are listed, with percentages in parentheses.

While all *Saimiri* share the same opsin alleles, the allele frequency distribution for *S. sciureus macrodon*, with a particularly high occurrence of AFT (53%) and a low occurrence of AFA (11%), differs from allele distributions of other *Saimiri* populations that have been studied (Table 3). Two previous analyses of *Saimiri* M/L opsin allele frequencies have employed pooled data from multiple *Saimiri* species sampled at different unspecified locations (Rowe & Jacobs, 2004), or from captive colonies of *S. boliviensis* of diverse geographic origins (Cropp et al., 2002). However, pooling data in this manner render interpretation difficult because it may obscure differences between local populations in different habitats. These considerations emphasize the importance of specifying the geographic origin of samples collected for genetic analyses.

Two other *Saimiri* populations with known geographic origins have been genotyped (Table 3). For *S*. *sciureus*, samples were collected from local populations being studied behaviorally in Guyana and Suriname ~2000 km east of Tiputini (Cropp et al., 2002). The allele frequency distribution of this eastern *Saimiri* is substantially different from that of the Tiputini population, with an AFA frequency 2.6 times as high and an AFT frequency only 70% as high ($\chi_{2}^{2}$ = 10.74, *p* =.005). This difference in opsin allele frequency distributions may be related to geographic differences in ecology, including intra‐ or interspecies competition (Boinski, 1999). Similar to the eastern *Saimiri*, the opsin allele distribution of the relatively isolated *S. oerstedii* population in Costa Rica differs from the Tiputini population in having a higher frequency of the AFA allele and a lower frequency of the AFT allele that may be ecologically relevant. However, the difference in the AFA frequency is not as great, being 1.8 times the frequency of the Tiputini sample, while the AFT allele is 69% as high ($\chi_{2}^{2}$ = 5.02, *p* =.081).

### 4.2 Visual ecology and intraspecific diversity of visual phenotypes

Each of the five taxa we investigated at Tiputini exhibited a diversity of inferred color vision phenotypes, reflecting the diversity of alleles in the population. Previous attempts to interpret the ecological relevance of opsin phenotype diversity have emphasized the different visual capabilities of dichromatic and trichromatic individuals in the context of intraspecific niche partitioning (Hogan et al., 2018; Melin et al., 2008, 2014, 2019; Veilleux et al., 2016). Naturalistic experiments (Caine et al., 2010; Saito et al., 2005; Smith et al., 2012), as well as modeling studies (De Araújo et al., 2006; Dominy & Lucas, 2001; Melin et al., 2014; Melin, Khetpal, et al., 2017; Osorio et al., 2004; Regan et al., 2001; Riba‐Hernández et al., 2004), have provided evidence that trichromats should have an advantage over dichromats in finding conspicuously colored yellowish‐reddish objects (e.g., fruits, flowers, young leaves) in a background of mature green foliage. Consistent with this expectation, Melin and colleagues found that trichromatic capuchins (*C. imitator*) in Costa Rica had higher intake rates of conspicuously colored fruits than dichromats (Melin, Chiou, et al., 2017) and also detected more small ephemeral flower patches (Hogan et al., 2018). By contrast, experimental and field studies suggest that dichromatic phenotypes are better at detecting camouflaged objects, such as insects (Caine et al., 2010; Melin et al., 2007; Smith et al., 2012; but see Abreu et al., 2019). These results have led researchers to suggest that the M/L opsin gene polymorphism facilitates mutual benefit of association between trichromatic and dichromatic individuals in a same foraging group or intraspecific niche divergence, wherein trichromats and dichromats forage on different food items and/or under different light conditions and achieve similar reproductive success (Fedigan et al., 2014; Melin et al., 2007, 2008; Mollon et al., 1984; Surridge et al., 2003; Veilleux et al., 2016).

Very little work has explored the performances of different color vision phenotypes within trichromacy and dichromacy. Most of the studies are model‐based, and they predict how different color vision phenotypes should perform in discriminating fruit against a background of green foliage. For example, trichromatic individuals with visual pigments more widely spectrally separated are predicted to have better discrimination on the red‐green chromatic axis than those with more closely spaced pigments, and thus to be better at detecting yellowish‐reddish fruits and flowers against a background of green foliage (De Araújo et al., 2006; Matsumoto et al., 2014; Melin et al., 2014; Osorio et al., 2004; Perini et al., 2009; Rowe & Jacobs, 2004). Indeed, the only field study of phenotype performance found that trichromatic capuchins with the largest spectral separation between their opsin alleles had the highest acceptance rates when foraging on reddish‐ripening figs (Melin et al., 2009), suggesting that individuals with this particular trichromatic phenotype may be better than other types of trichromats in evaluating ripeness and palatability.

Model predictions, however, depend on the fruits included in the sample. For trichromats, Melin et al. (2014) predicted that female capuchins with the more red‐shifted trichromatic phenotype (545/561 nm) should have the best detection performance for preferred and heavily consumed foods, while the phenotype with the greatest spectral separation (532/561 nm) should have the best detection performance for seasonally critical foods. For males and for female dichromats, they predicted that the SYT dichromats should have the highest performance in detecting yellowish‐reddish fruits against green foliage, while the AFA dichromats should have the lowest performance on this discrimination, but instead, be better at detecting bluish fruits (Melin et al., 2014; Osorio et al., 2004). Field studies have not yet been able to test these predictions of the foraging performance of different dichromat phenotypes.

#### 4.2.1 Visual ecology of the atelids

Both *Ateles* and *Lagothrix* rely heavily on ripe fruit in their diets (Dew, 2005; Di Fiore, 2004; Di Fiore et al., 2008; Link & Di Fiore, 2006; Stevenson et al., 1994), which suggests strong selective pressure for detecting ripe fruit compared with other primate species. For these highly frugivorous atelids, the absence of the AFA allele limits the spectral separation that is possible for trichromats and may imply a limitation to red‐green discrimination important for detecting reddish ripe fruits. However, this limitation is partially offset by enlargement of the spectral separation of the SYT and either the SFT (*Ateles*) or AFT (*Lagotrhix*) alleles by the substitutions Y213D and N294K at other sites in the protein (Matsumoto et al., 2014). The spectral separation of the M and L pigments is 15 nm for *Ateles* and 19 nm for *Lagothrix*. Although these are smaller than the spectral separation of some trichromatic phenotypes of the non‐atelids (Table 2), a previous behavioral experiment shows that the 15‐nm spectral separation is still sufficient in discriminating red‐green color contrast (Saito et al., 2005). At the population level, the lack of an AFA allele, along with the high frequency of the long‐wavelength SYT allele, may be adaptive because it results in a high proportion of dichromats with the SYT allele (Kawamura, 2018), which are predicted to be better than AFT or SFT dichromats at detecting yellowish‐reddish ripe fruits (Melin et al., 2014; Osorio et al., 2004).

Field studies of atelid visual ecology are limited and thus far have failed to detect foraging differences among color vision phenotypes. For example, a study of *A. geoffroyi* at Sector Santa Rosa, a dry forest in Costa Rica, found no differences in foraging efficiency at short range between dichromats and trichromats (Hiramatsu et al., 2008, 2009). Given this result, Hiramatsu, Melin et al. hypothesized that trichromacy may provide a greater advantage for long‐distance detection of yellowish‐reddish resources (particularly small and/or ephemeral patches), while luminance cues and olfactory cues may be more salient for short‐range fruit detection (Hiramatsu et al., 2008, 2009; Melin et al., 2014). Currently, there has been no study relating color vision phenotype to foraging ecology in *A. belzebuth* or any *Lagothrix* species. While the Tiputini *A. belzebuth* and the Santa Rosa *A. geoffroyi* share the same vision phenotypes with high proportion of SYT dichromats, Tiputini represents a vastly different floristic environment with a larger community of sympatric primates and other frugivores. Hence, the potential competitive situation is quite different. Moreover, *Lagothrix* seasonally consumes a substantial amount of animal prey (Dew, 2005; Di Fiore, 1997, 2004), which adds another dimension to its visual ecology. An analysis of the foraging performance of different visual phenotypes of these two atelids at Tiputini would be valuable for understanding the evolution and maintenance of polymorphic trichromacy in these genera.

#### 4.2.2 Visual ecology of *Plecturocebus*, *Pithecia*, and *Saimiri*

In our non‐atelid taxa, the larger number of segregating M/L alleles is accompanied by a greater diversity of visual phenotypes, including more types of dichromats and trichromats with peak sensitivities in different wavelength regions. This greater diversity of visual phenotypes may reflect a sensory adaptation for greater dietary diversity, including increased consumption of animal prey and/or seeds (Bicca‐Marques & Heymann, 2013; Charpentier et al., 2015; Lopes, 2016; Montague, 2011). In contrast to the atelids, only 17% (*Plecturocebus*), 20% (*Pithecia*), and 34% (*Saimiri*) of non‐atelid dichromats carried the long‐wavelength SYT allele best suited for detecting yellowish‐reddish fruits. Instead, 50%–60% of these dichromats carried the mid‐wavelength (545 nm) AFT allele. A small‐to‐moderate percentage of dichromats carried the short wavelength (532 nm) AFA allele, predicted to be the best for detecting bluish objects (Melin et al., 2014; Osorio et al., 2004): *Plecturocebus*, 17%; *Pithecia*, 20%; and *Saimiri*, 7%. We note that the colors of plant foods consumed by *Saimiri* at Tiputini generally covary with opsin allele frequencies; most plant taxa consumed were yellow/green (48%) or yellow‐red (44%) rather than bluish (<8%; Montague, 2011), mirroring the higher frequency of the mid‐wavelength AFT allele and the low frequency of the short‐wavelength AFA allele in the *Saimiri* population. Similar data on food color are not yet available for *Plecturocebus* or *Pithecia* at Tiputini.

The greater opsin and dietary diversity within the non‐atelid taxa may facilitate intraspecific partitioning of “visual niche space,” permitting individuals within a social group to reduce intraspecific competition by feeding on different resources. At other study sites, for example, dichromats are more efficient than trichromats at capturing camouflaged insects (Melin et al., 2007, 2010; Smith et al., 2012). There is currently little evidence that different dichromatic phenotypes vary in detecting different types of insects or fruits (e.g., Abreu et al., 2019), and further study will be important for understanding the ecology and evolution of opsin genotypes.

For each of the three non‐atelid taxa, we identified three different types of trichromats with differing degrees of spectral separation between the mid‐ and long‐wavelength alleles (Table 2). Surprisingly, for *Plecturocebus*, spectral separation of the alleles was relatively small for two of the three trichromatic phenotypes (5 and 10 nm), which may result in poor red‐green color discrimination. This result is particularly interesting given the presence of four alleles in the population. The evolution of these spectrally similar alleles may reflect a relaxation of selection for acute color discrimination or the increased use of other sensory cues, such as shape, brightness, odor, or touch, to acquire essential foods. Future work should explore the sensory characteristics of *Plecturocebus* foods and signatures of selection on the M/L opsin gene in this population to investigate these possibilities.

For *Pithecia* and *Saimiri*, all trichromats had spectral separations roughly comparable to or greater than those characterizing the frugivorous atelids (*Ateles* and *Lagothrix*: 15–19 nm; *Pithecia* and *Saimiri*: 13–28 nm). Thus, the AFA/SYT trichromats of the seed predator *Pithecia* and the insectivore/frugivore *Saimiri* should theoretically perform better than the atelids at detecting ripe red‐yellow fruits against a background of green foliage. For reference, we note that the color vision of human trichromats is based on a 22–29 nm spectral separation of visual pigments (Merbs & Nathans, 1992), similar to the widest spectral separation seen in our sample of neotropical primates. Yet in *Saimiri*, the only species for which sample size was sufficient to compare the frequency of trichromatic phenotypes, only 16% of trichromats had the widest spectral separation (26 nm). Instead, well over half of *Saimiri* trichromats (61%) exhibited a more red‐shifted trichromatic phenotype (545/558 nm), suggesting that individuals with different trichromatic phenotypes may differ in resources that they are best able to detect, a possibility that has not yet been tested empirically in any field study.

Overall, the diversity of dichromatic and trichromatic phenotypes observed in these sympatric species offers multiple possibilities for intraspecific niche partitioning. Visual diversity may enable individual members of foraging groups to exploit somewhat different resources, thus reducing within‐group competition, broadening the available resource base (Hogan et al., 2018; Melin et al., 2007, 2010; Smith et al., 2012), and creating a mutual benefit to group members (Veilleux et al., 2016). Individuals with different visual phenotypes may also be able to better detect different predators (Pessoa et al., 2014) or have other perceptual advantages that could contribute to overall fitness. Further studies of the behavior of specific color vision phenotypes are needed to clarify how different phenotypes utilize “visual niche space.”

### 4.3 Intraspecific variation, interspecific variation, and niche partitioning

At Tiputini, each atelid species had one type of trichromat and two types of dichromats, while each of the non‐atelids exhibited three or four types of dichromats and three types of trichromats. The implications of these results for feeding ecology and niche partitioning depend upon the interactions of visual phenotypes with dietary preferences, anatomical and physiological adaptations, and environmental factors for each species. However, the predicted differences in performance among color vision phenotypes for different food objects provide potential mechanisms for both intraspecific and interspecific niche partitioning.

The greater diversity of opsin genotypes and the relatively low frequency of SYT dichromats among non‐atelids at Tiputini may represent a form of interspecific partitioning of visual niche space. *Ateles* and *Lagothrix*—which primarily carry the SYT dichromatic phenotype—are large‐bodied primates, and they can readily displace other primate species when competing for ripe fruit (D.M. Snodderly & A. Di Fiore, unpublished observations 2015‐2019). Thus, interspecific feeding competition may exert selective pressure on the non‐atelid taxa to exploit food resources that are better detectable by non‐SYT phenotypes, which would subsequently influence the allele frequency distribution of the M/L opsin gene in those taxa. Indeed, all three non‐atelid species exhibit a greater dietary diversity than the atelids with less dependence on ripe fruit and greater consumption of animal prey and seeds (Bicca‐Marques & Heymann, 2013; Charpentier et al., 2015; Montague, 2011).

Questions about intraspecific variation and “visual niche space” are particularly interesting to consider for sites like Tiputini with rich biodiversity, large primate communities, and a host of other mammalian and avian species that also forage in the forest canopy. We identified substantial variation between sympatric species, as well as variation between populations at our site and congeners at other sites. We suggest that the differences in opsin allele frequencies between *Saimiri* at Tiputini and *Saimiri* at other sites may reflect niche partitioning with different sets of sympatric species.

Allele frequency data for the same primate species in different habitats are rare, but they are available for two *Cebus* populations in northwestern Costa Rica. At Sector Santa Rosa, where *Cebus* is found sympatrically with *A. geoffroyi* and *Alouatta palliata*, allele frequencies were 20.6% for AFA (530 nm), 36% for AFT (545 nm), and 56.1% SYT (560 nm; Melin et al., 2014). However, *Ateles* is not present at the Lomas Barbudal Biological Reserve, likely due to human hunting (Chapman et al., 1989), and interestingly, allele frequencies in the *Cebus* population were substantially different: 41.4% AFA, 13.8% AFT, and 44.8% SYT (Vogel et al., 2007). It is intriguing to speculate that the loss of a major competitor and/or other environmental differences led to a shift in the “visual niche space” occupied by *Cebus* at Lomas Barbudal, leading to a concomitant change in allele frequencies.

## 5 CONCLUSIONS

Given the renewed interest in the effects of within‐species variation on interspecific niche partitioning (Bolnick et al., 2011; Violle et al., 2012), opsin gene polymorphisms among neotropical primates provide an exciting and rich system to investigate these questions. In this study, we surveyed M/L opsin diversity in five of the ten primate species at the Tiputini Biodiversity Station in Amazonian Ecuador, offering the most extensive survey so far of opsin diversity across a neotropical primate community. We found the first evidence of four opsin alleles in a wild *Plecturocebus* population and substantial interspecific and intraspecific variation in opsin allele and genotype frequencies among other sympatric taxa. Our results highlight a need to understand the role of different vision phenotypes in foraging efficiency and detection performance for different foods consumed by each primate species. We conclude that a deeper understanding of opsin gene diversity and foraging ecology will shed light on niche partitioning, flexibility, and resilience in neotropical primates.

## CONFLICT OF INTEREST

None declared.

## AUTHOR CONTRIBUTIONS

**Carrie C. Veilleux:** Conceptualization (equal); Data curation (equal); Formal analysis (equal); Investigation (equal); Methodology (equal); Project administration (lead); Supervision (equal); Visualization (lead); Writing‐original draft (lead); Writing‐review & editing (equal). **Shoji Kawamura:** Conceptualization (equal); Data curation (equal); Formal analysis (equal); Investigation (equal); Methodology (equal); Resources (equal); Supervision (equal); Writing‐review & editing (equal). **Michael J. Montague:** Conceptualization (equal); Data curation (equal); Formal analysis (equal); Funding acquisition (equal); Investigation (equal); Methodology (equal); Writing‐review & editing (equal). **Tomohide Hiwatashi:** Investigation (equal); Methodology (equal). **Yuka Matsushita:** Data curation (equal); Formal analysis (equal); Investigation (equal); Methodology (equal). **Eduardo Fernandez‐Duque:** Funding acquisition (equal); Investigation (equal); Writing‐review & editing (equal). **Andres Link:** Investigation (equal); Methodology (equal); Writing‐review & editing (equal). **Anthony Di Fiore:** Conceptualization (equal); Data curation (equal); Formal analysis (equal); Funding acquisition (equal); Methodology (equal); Project administration (supporting); Resources (equal); Supervision (equal); Writing‐original draft (supporting); Writing‐review & editing (equal). **D. Max Snodderly:** Conceptualization (equal); Formal analysis (equal); Funding acquisition (equal); Project administration (equal); Resources (equal); Supervision (equal); Writing‐original draft (lead); Writing‐review & editing (equal).

## Supporting information

Supplementary Material

## Data Availability

DNA sequences for exons 3 and 5 of the M/LWS opsin gene for *Plecturocebus* and *Pithecia* individuals have been deposited in GenBank (accession numbers: MT984248–MT984263 and MT995856–MT995889; see Supporting Information for accession numbers by individual and species).
