# Supplementary material for: Color vision and niche partitioning in a diverse neotropical primate community in lowland Amazonian Ecuador
Source: Ecol Evol. 2021 Mar 30;11(10):5742–58. doi: 10.1002/ece3.7479 (PMC8131790; doi:10.1002/ece3.7479)
Supplement: Supplementary file 1 — Supplementary Material [file ECE3-11-5742-s001.docx]

**Supporting Information**

**Color vision and niche partitioning in a diverse neotropical primate community in lowland Amazonian Ecuador**

Carrie C. Veilleux, Shoji Kawamura, Michael J. Montague, Tomohide Hiwatashi, Yuka Matsushita, Eduardo Fernandez-Duque, Andres Link, Anthony Di Fiore, D. Max Snodderly

Contents

[Materials and Methods S1: PCR conditions 2](#_Toc52538515)

[Materials and Methods S2: RT-PCR conditions for SNP genotyping experiments 2](#_Toc52538516)

[Table S1. Primers for *Pithecia* and *Plecturocebus* PCRs. 3](#_Toc52538517)

[Table S2. Primers for *Ateles* and *Lagothrix* PCRs. 3](#_Toc52538518)

[Table S3. Sequence accession numbers by individual and species for *Plecturocebus* and *Pithecia*. 4](#_Toc52538519)

[References 5](#_Toc52538520)

## **Materials and Methods S1: PCR conditions**

For *Plecturocebus* and *Pithecia* samples, exons 3 and 5 were amplified in polymerase chain reactions (PCRs) using the primers in Table S1. Each PCR contained 3 μL DNA template, 14.4 μL ddH_2_O, 5 μL taq 5x green Go-Taq Flexi Buffer (Promega), 1.5 μL MgCl_2_ (25mM), 0.5 μL 10μM dNTPs, 0.5 μL 20μM forward primer, 0.5uL 20μM reverse primer, and 0.13 μL Go-Taq DNA Polymerase (Promega), for a total of 25.5 μL reaction. The PCRs were carried out with the following conditions: (1) initial denaturation at 95°C for 2 minutes; (2) thirty-five cycles of 30 seconds at 95°C, 30 seconds at 57°C, 1 minute at 72°C, and (3) final extension at 72°C for 1 minute.

For *Ateles* and *Lagothrix* samples, exons 3 and 5 were amplified following Matsumoto et al. (2014).

## **Materials and Methods S2: RT-PCR conditions for SNP genotyping experiments**

Genotyping experiments were carried out in 10μL reactions with 1.0 μL of genomic DNA extract from feces, 5.0 μL of TaqMan® Universal PCR Master Mix (containing dNTP’s, AmpliTaq Gold® DNA polymerase, and reaction buffer), 3.75 μL of ddH_2_O, and 0.25 μL of the 40X Assay Mix. Reactions were carried out in duplicate using the following thermal cycling conditions: 1) a pre-PCR read hold at 60°C for 30 seconds; 2) an initial hold at 95°C for 10 minutes; 3) 40 cycles of a 15 second denaturing step at 95°C followed by a 60 second annealing and extension step at 60°C. These steps were followed by a post-PCR hold at 60° for 30 seconds whereby the automated genotype designations were performed. Finally, in order to ensure a standard-sized amplicon across all sample wells, a melt curve analysis was carried out with 81 cycles of 0.5°C increments every 10 seconds beginning at 55°C.

Automated genotype calls were made using the TaqMan® Genotyper Software (Applied Biosystems). The TaqMan® SNP-typing process was replicated to validate every homozygous and heterozygous genotype at least twice. Additional analyses of sex and parentage using multilocus SSR genotypes from replicate fecal samples were used to confirm individual identity and expected patterns of opsin inheritance within social groups (Montague, Disotell, & Di Fiore, 2014).

## **Table S1. Primers for *Pithecia* and *Plecturocebus* PCRs.**

| **Primer** | **Sequence** | **Reference** |
| --- | --- | --- |
| *Exon 3* |  |  |
| Forward primer | CTC TGG TCC CTG GCC ATC ATT | Corso et al. (2016) |
| Reverse primer | CCC CTT ACC TGC TCC AAC CAA | Corso et al. (2016) |
|  |  |  |
| *Exon 5* |  |  |
| Forward primer | GAA TCC ACC CAG AAG GCA G | designed in Geneious |
| Reverse primer | GGG GTT GTA GAT AGT GGC A | designed in Geneious |

## **Table S2. Primers for *Ateles* and *Lagothrix* PCRs.**

| **Primer** | **Sequence** | **Reference** |
| --- | --- | --- |
| *Exon 3* |  |  |
| Forward primer | CCTCCTGATTTTGAAAGCTGTCAGCTGG | Matsumoto et al. (2014) |
| Reverse primer | CAGAGGGGCCCAGAGAAAGGAATGATTT | Matsumoto et al. (2014) |
|  |  |  |
| *Exon 4* |  |  |
| Forward primer | ACTGGCTGCCGGCCCTTCTCTCCAG | Matsumoto et al. (2014) |
| Reverse primer | AGGCCAGGAGGGATCGGGGGCTTAC | Matsumoto et al. (2014) |
|  |  |  |
| *Exon 5* |  |  |
| Forward primer | TATGCCTGGGTCACCTGCCTCTT | Matsumoto et al. (2014) |
| Reverse primer | TCAGAGACACGACTCCAGGTGGA | Matsumoto et al. (2014) |

## **Table S3. Sequence accession numbers by individual and species for *Plecturocebus* and *Pithecia*.**

| **Species** | **Individual** | **Sex** | **Exon 3** | **Exon 5** |
| --- | --- | --- | --- | --- |
| *Plecturocebus discolor* | Liam | M | MT984248 | MT995856 |
|  | Baleia | F | MT984249 | MT995857 |
|  | Banana | F | MT984250 | MT995858 |
|  | Bandito | M | MT984251 | MT995859 |
|  | Bongo | M | MT984252 | MT995860 |
|  | Bramble | F | MT984253 | MT995861 |
|  | Buttercup | F | MT984254 | MT995862 |
|  | Homero | M | MT984255 | MT995863 |
|  | Huito | M | MT984256 | MT995864 |
|  | Kebac | M | MT984257 | MT995865 |
|  | Kia | F | MT984258 | MT995866 |
|  | Kong | M | MT984259 | MT995867 |
|  | Luciferus | M | MT984260 | MT995868 |
|  | Lulu | F | MT984261 | MT995869 |
|  | Puma | F | MT984262 | MT995870 |
|  | Saul | M | MT984263 | MT995871 |
|  |  |  |  |  |
| *Pithecia aequatorialis* | Dharma | F | MT995872 | MT995881 |
|  | Morpho | M | MT995873 | MT995882 |
|  | Marigold | F | MT995874 | MT995883 |
|  | Mayer | M | MT995875 | MT995884 |
|  | Mona | F | MT995876 | MT995885 |
|  | Mondika | M | MT995877 | MT995886 |
|  | Pipian | M | MT995878 | MT995887 |
|  | Sancho | M | MT995879 | MT995888 |
|  | Milo | F | MT995880 | MT995889 |

## **References**

Corso, J., Bowler, M., Heymann, E. W., Roos, C., & Mundy, N. I. (2016). Highly polymorphic colour vision in a New World monkey with red facial skin, the bald uakari (*Cacajao* *calvus*). *Proc. R. Soc. B*, *283*(1828), 20160067. doi: 10.1098/rspb.2016.0067

Matsumoto, Y., Hiramatsu, C., Matsushita, Y., Ozawa, N., Ashino, R., Nakata, M., … Kawamura, S. (2014). Evolutionary renovation of L/M opsin polymorphism confers a fruit discrimination advantage to ateline New World monkeys. *Mol Ecol*, *23*, 1799–1812. doi: 10.1111/mec.12703

Montague, M. J., Disotell, T. R., & Di Fiore, A. (2014). Population genetics, dispersal, and kinship among wild squirrel monkeys (*Saimiri* *sciureus macrodon*): preferential association between closely related females and its implications for insect prey capture success. *Int J Primatol*, *35*, 169–187. doi: 10.1007/s10764-013-9723-7
